# Comparative Pathogenomics of *Aeromonas veronii* from Pigs in South Africa: Dominance of the Novel ST657 Clone

**DOI:** 10.3390/microorganisms8122008

**Published:** 2020-12-16

**Authors:** Yogandree Ramsamy, Koleka P. Mlisana, Daniel G. Amoako, Akebe Luther King Abia, Mushal Allam, Arshad Ismail, Ravesh Singh, Sabiha Y. Essack

**Affiliations:** 1 Medical Microbiology, College of Health Sciences, University of KwaZulu-Natal, Durban 4000, South Africa; Singhra@ukzn.ac.za; 2 National Health Laboratory Service, Durban 4001, South Africa; koleka.mlisana@nhls.ac.za; 3 Antimicrobial Research Unit, College of Health Sciences, University of KwaZulu-Natal, Durban 4000, South Africa; amoakodg@gmail.com (D.G.A.); lutherkinga@yahoo.fr (A.L.K.A.); essacks@ukzn.ac.za (S.Y.E.); 4 Sequencing Core Facility, National Institute for Communicable Diseases, National Health Laboratory Service, Johannesburg 2131, South Africa; MushalA@nicd.ac.za (M.A.); ArshadI@nicd.ac.za (A.I.)

**Keywords:** genomics, *Aeromonas veronii*, intensive pig farming, abattoir, antibiotics, mobile genetic elements, global phylogeny

## Abstract

The pathogenomics of carbapenem-resistant *Aeromonas veronii (A. veronii)* isolates recovered from pigs in KwaZulu-Natal, South Africa, was explored by whole genome sequencing on the Illumina MiSeq platform. Genomic functional annotation revealed a vast array of similar central networks (metabolic, cellular, and biochemical). The pan-genome analysis showed that the isolates formed a total of 4349 orthologous gene clusters, 4296 of which were shared; no unique clusters were observed. All the isolates had similar resistance phenotypes, which corroborated their chromosomally mediated resistome (bla*_CPHA_*_3_ and bla*_OXA-_*_12_) and belonged to a novel sequence type, ST657 (a satellite clone). Isolates in the same sub-clades clustered according to their clonal lineages and host. Mobilome analysis revealed the presence of chromosome-borne insertion sequence families. The estimated pathogenicity score (P_score_ ≈ 0.60) indicated their potential pathogenicity in humans. Furthermore, these isolates carried several virulence factors (adherence factors, toxins, and immune evasion), in different permutations and combinations, indicating a differential ability to establish infection. Phylogenomic and metadata analyses revealed a predilection for water environments and aquatic animals, with more recent reports in humans and food animals across geographies, making *A. veronii* a potential One Health indicator bacterium.

## 1. Introduction

*Aeromonas* spp. are Gram-negative, rod-shaped, non-sporulating, non-acid-fast, and facultative anaerobic bacteria that have been recognized as emerging nosocomial pathogens [1]. They belong to the family Aeromonadaceae, class Gammaproteobacteria that encompasses three genera, viz., *Tolumonas*, *Oceanimonas* and *Aeromonas* [2,3]. *Aeromonas* share many similar biochemical characteristics with *Enterobacterales* but are easily differentiated, with *Aeromonas* being oxidase-positive [4]. The *Aeromonas* genus has a complex, dynamic taxonomy due to the expanding number of new species and its high intra- and interspecies genetic variability [5,6]. This genus currently comprises 36 species, with *A. dhakensis*, *A. hydrophila*, *A. caviae*, *A. salmonicidai*, and *A. veronii* being the most clinically relevant and pathogenic species [3,7,8].

*Aeromonas* spp. have been implicated in a range of diseases in animals and humans, including gastroenteritis, septic arthritis, peritonitis, osteomyelitis, myositis, ocular infections, meningitis, cholangitis, pneumonia, hemolytic uremic syndrome, and urinary tract infections [9,10,11]. Members of this genus are widely distributed across numerous ecosystems. *A. veronii* has been isolated from the environment (air, water and soil), food animals (shellfish, poultry, cattle, and pigs), as well as various human infections, making it a potential One Health indicator pathogen [5,12,13,14,15]. Faecal carriage rates of *Aeromonas* in normal humans are thought to be between 0% and 4% [16], while carriage rates in patients with symptomatic diarrhea range between 0.8 and 7.4 [17].

The virulence and pathogenesis of *Aeromonas* have been described as multifaceted and linked to the expression of genes that encode different metalloproteins, secretion systems, structural components, and toxins [4,18,19,20]. Studies have also reported the expression of different immune-related genes in the host, following an *Aeromonas* infection, including those involved in apoptosis, cell signaling, and pathogen recognition [3,21]. Although these virulence factors may confer variable abilities to establish infection, knowledge of *A. veronii* pathogenicity is currently incomplete. Also, despite the apparent increase in the incidence of this emerging pathogen globally, information on *A. veronii* in humans, animals, and the environment in Africa is limited. Thus, in this study, we describe for the first time in Africa, the comparative genomics of *A. veronii* recovered from pigs in KwaZulu-Natal, South Africa.

## 2. Materials and Methods

### 2.1. Ethical Approval

This study was approved by the Animal Research Ethics Committee (AREC/079/018D), University of KwaZulu-Natal (UKZN, Durban, South Africa). Permission for animal sampling was obtained from the Department of Agriculture, Forestry and Fisheries, Republic of South Africa (Ref 12/11/15). The State Veterinarian and the Health and Safety Officers at the abattoir were duly consulted. All samplers took a compulsory competency to ensure that all health and safety standards were met, and the necessary precautions were taken during animal sampling.

### 2.2. Sampling

This study was part of a larger point-prevalence study undertaken to determine the molecular epidemiology of carbapenem-resistant bacteria in humans (from public hospitals), food animals (a food processing plant with an abattoir), and the environment (a wastewater treatment plant) in uMgungundlovu, KwaZulu-Natal, South Africa. The current study focused on pigs. A total of 345 rectal swabs were collected from pigs, post-slaughter at an abattoir, using nylon-flocked swabs. Swabs were transported in 5 mL of Amies gel transport medium on ice to a reference laboratory authorized to process animal samples and processed within 6 h of sample collection.

### 2.3. Isolation and Identification

#### 2.3.1. Screening of Carbapenemase-Producing Isolates

All rectal swabs were cultured on ChromID CARBA chromogenic agar (BioMérieux, Marcy l’Étoile, France) as described previously [22,23], with an incubation period of 18 to 24 h at 37 °C. Carbapenemase-negative *Klebsiella pneumoniae* ATCC 700603 and carbapenemase-positive *Klebsiella pneumoniae* ATCC BAA-1705 served as controls.

#### 2.3.2. Confirmation of Isolates and Determination of Antibiotic Susceptibility Profiles

The carbapenem-resistant isolates obtained were sub-cultured onto MacConkey plates and subjected to phenotypic identification and susceptibility testing using the VITEK 2 (BioMérieux, Marcy l’Étoile, France) automated platform. The Clinical and Laboratory Standards Institute (CLSI) interpretative criteria were used to categorize isolates as susceptible or resistant [24]. The following β-lactam antibiotics were tested: ampicillin, amoxicillin, amoxicillin-clavulanate, ceftriaxone, ceftriaxone, cefepime, cefuroxime, cefoxitin, ceftazidime, imipenem, meropenem and piperacillin-tazobactam.

### 2.4. DNA Purification, Genome Sequencing and Pre-Processing of Sequence Data

Isolates that were confirmed as *Aeromonas* were sub-cultured on nutrient agar (Sigma-Aldrich, St. Louis, MO, USA) at 37 °C for 24  h. Genomic DNA was then extracted from these pure cultures using the Quick-DNA™ Bacterial Miniprep Kit (Inqaba Biotechnical Industries (Pty) Ltd., Pretoria, South Africa). The extracted DNA was quantified using a Nanodrop 8000, Qubit (Thermo Scientific, Waltham, MA, USA) and verified on an agarose gel electrophoresis. A paired-end library (2 × 300 bp) was prepared using Illumina Nextera XT DNA Sample Preparation Kit and sequenced on a MiSeq machine (Illumina, San Diego, CA, USA). The generated sequenced reads were quality assessed, trimmed, and de novo assembled using the SKESA Assembler (version 2.3; https://github.com/ncbi/SKESA), with default parameters for all software, unless otherwise specified.

### 2.5. Genome Visualization and Annotation

The genomes of the isolates were visualized using the GView Server [25]. The National Center for Biotechnology Information (NCBI) Prokaryotic Genome Annotation Pipeline (PGAP; version 4.3) [26], and Rapid Annotation using Subsystem Technology (RAST) Server (version 2.0) [27] were used for annotation of the size, GC content, number of contigs, N50, L50, number of RNAs, protein-coding sequences for subsystem categorization, and comparison of the isolates. The OrthoVenn2 web server [28] was used to identify orthologous gene clusters that were either unique or shared among *A. veronii* strains. The analysis was performed with default parameters for the protein-coding genes of the strains.

### 2.6. Whole Genome Sequencing-Based Confirmation and Molecular Typing of *Aeromonas Veronii*

The generated contigs from the WGS data were used to confirm the identity of the isolates on the Pathogenwatch platform [29]. The assembled genomes were submitted to the *Aeromonas* MLST database, which assessed the allelic profiles of six housekeeping genes to assign the new sequence type (ST) [30]. An eBURST [31] algorithmic analysis was performed in the MLST database (https://pubmlst.org/aeromonas/) to ascertain whether the novel ST was a single-locus variant (SLV) or double-locus variant (DLV) of the known STs. The STs of globally deposited *A. veronii* genomes were obtained from the PATRIC database (https://www.patricbrc.org/view/Taxonomy/2).

### 2.7. Antibiotic Resistance Genes, Efflux Genes, and Mobile Elements Identification

The bacterial analysis pipeline of GoSeqIt via ResFinder [32], Antibiotic Resistance Gene-Annotation database (ARG-ANNOT) [33], and the Comprehensive Antibiotic Resistance Database (CARD) [34] tools were also used to annotate and identify resistance and efflux genes. PHAge Search Tool [35] server was used for the identification, annotation, and visualization of prophage sequences. The presence of insertion sequences and transposons was determined by blasting contigs on the ISFinder database [36], while the presence of integrons was ascertained from the PGAP and RAST subsystems and blasted on the IntegronFinder database. The distribution of CRISPR-Cas systems in *A*. *veronii* genomes was determined by CRISPRfinder [37]. Annotations from the Restriction-Modification Finder predicted the R-M system in the isolates [38].

### 2.8. Assessment of Pathogenic Potential and Prediction of Putative Virulence Factors

The pathogenic potential of the *A. veronii* isolates was assessed using the PathogenFinder web service under the automated mode [39]. All isolates were subjected to the pathogenicity prediction using Fasta formatted genome data. Virulence determinants (sequences and functions), corresponding to four major bacterial virulence factors (adherence, motility, secretion system and toxin) associated with *A. veronii*, were searched for in the pathogenic bacteria database, VFanalyzer [40] and validated by blasting assembled genomes to a pseudomolecule generated by concatenating a set of target genes using the NCBI in-house BLASTN tool. The known *A. veronii* B565 (4,551,783 bp, Accession number: NC_015424) was used as the reference genome.

### 2.9. Global Phylogenomic Relationship and Metadata Analysis

A phylogenomic analysis was undertaken to compare the genomes of the study isolates with all available *A. veronii* genomes downloaded from GenBank and PATRIC via CSI Phylogeny-1.4 [41]. The genome of *Tolumonas auensis* DSM 9187 (GenBank accession number: CP001616) of the Aeromonadaceae family was used to root the tree, facilitating the configuration of the phylogenetic distance between the isolates on the branches. A bootstrapped indicator with 100 replicates was applied to identify recombined regions and provide the phylogenetic accuracy in groups with little homoplasy. Figtree (http://tree.bio.ed.ac.uk/software/figtree/) was used to visualize and edit the phylogenetic tree. The phylogeny was visualized alongside annotations for isolate metadata (WGS in silico molecular typing, source, and country) using Phandango [42], to provide a comprehensive analysis of the generated phylogenomic tree.

### 2.10. Data Availability

The raw read sequences and assembled whole-genome contigs have been deposited in GenBank. The data is available under project number PRJNA564235.

## 3. Results

### 3.1. Prevalence and Phenotypic Resistance Profiles of A. Veronii Isolates

Five (5) carbapenem-resistant (CR) *A. veronii* isolates were obtained from 5 different pig samples, giving a carriage rate of 1.5% (5/345) among the sampled pig population. The isolates exhibited similar resistance phenotypes, all of them displaying 100% resistance to ampicillin, amoxicillin and imipenem, except for piperacillin-tazobactam to which only one isolate showed resistance (Table 1).

### 3.2. Genome Confirmation, Statistics, Annotation, and Visualization

**Figure 1:**
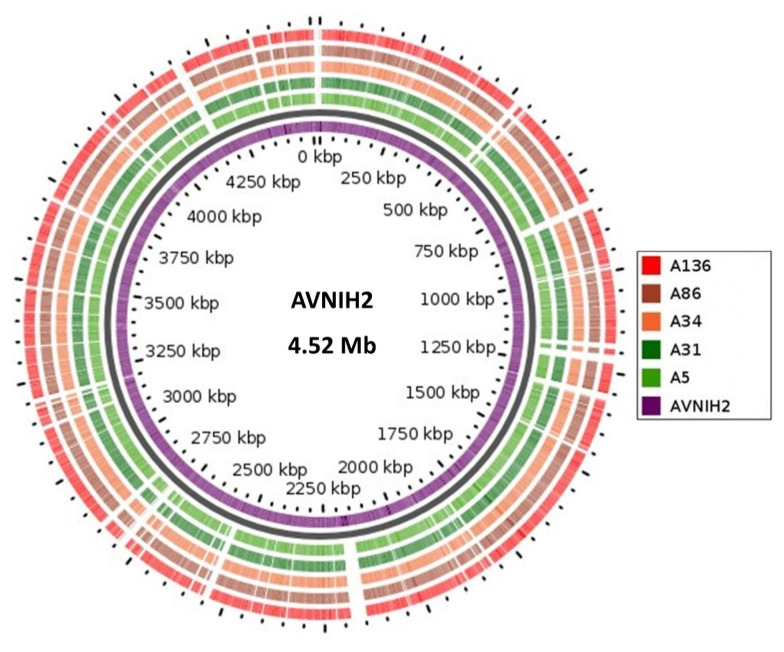
Comparative visualization of the isolates (*n* = 5) with the *A. veronii* reference strain (AVNIH2, Accession number: LRBO00000000). The map was constructed using the GView online server (https://server.gview.ca/). The concentric circles represent comparisons between AVNIH2 and, starting with the inner circle, genome assemblies from *A. veronii* genomes (isolate ID: A5, A31, A34, A86 and A136). Colour codes are given for each isolate with a synteny identity, ranging from >98%.

The global Pathogenwatch platform confirmed the phenotypic identity of the *A. veronii* genomes. The genomic features of the sequences, in terms of size, GC content, number of contigs, N50, L50, number of RNAs and number of protein-coding sequences are listed in Supplementary Materials Table S1. The genome size of the *A. veronii* isolates ranged from 4.64 Mb to 4.77 Mb, with GC content of 58.2–58.5 and a coverage range between 99% to 102%. Comparative visualization analysis, via the GView server (Figure 1), showed a similarity of DNA synteny with >98% coverage and identity from the AVNIH2 reference in all the *A. veronii* genomes.

**Figure 2:**
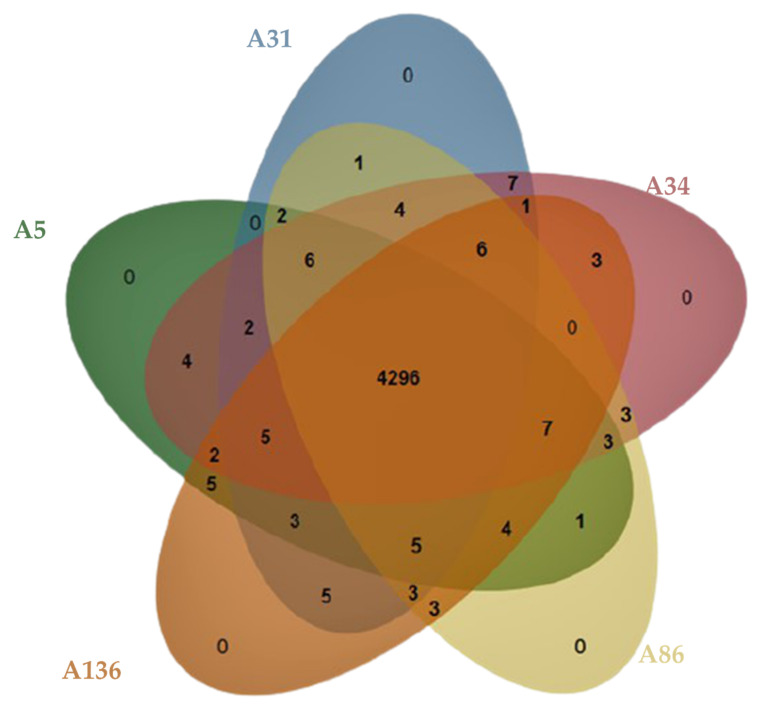
Venn diagram of the unique and shared number of orthologous gene clusters among the genomes of the five *A. veronii* isolates.

**Figure 3:**
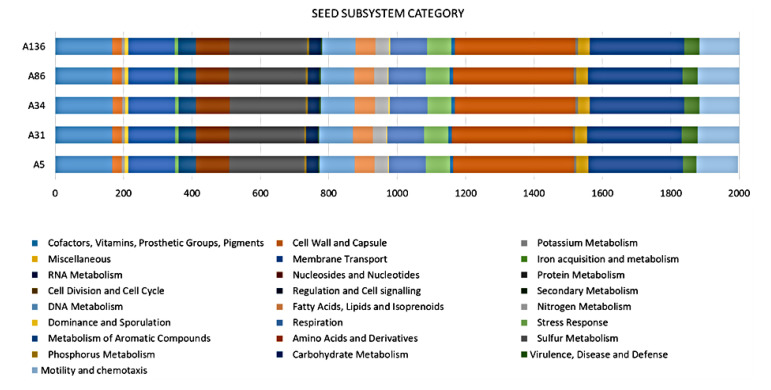
SEED subsystem category for *A*. *veronii* genomes. Comparison of functional categories in 5 *A*. *veronii* genomes based on SEED. The functional categorization is based on the roles of annotated and assigned genes. Each coloured bar represents the number of genes assigned to each category.

Pan-genome and ortholog analysis revealed that all the isolates formed a total of 4349 clusters and shared 4296 orthologous clusters (98.8%) (Figure 2). The singletons ranged from 10 to 42 gene clusters. The isolates shared a similar range of orthologous clusters (*n*_range_= 26–30) with each other; however, no unique orthologue gene cluster (*n* = 0) was found (Figure 3).

### 3.3. Defence Systems (CRISPRCas Cluster and Restriction-Modification (R-M) System), Antibiotic Resistome, Mobilome and Genetic Environment Analysis

**Figure 4:**
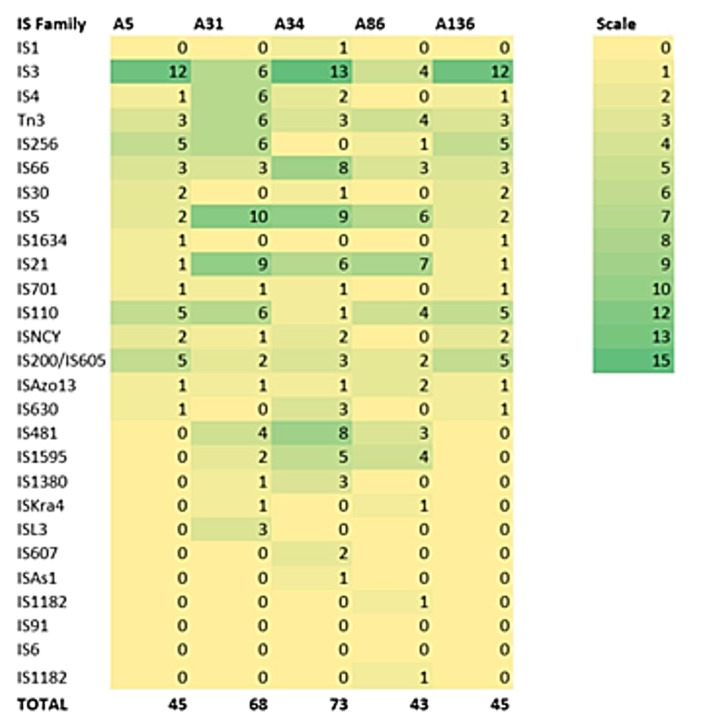
Distribution of insertion elements in *A. veronii* genomes. The total number of insertion sequences includes complete, partial, and unknown regions.

The isolates shared a similar resistome (same resistance and efflux genes), with the mobilome comprised of variable insertion sequence families (Table 2 and Figure 4), while plasmids, integrons and intact prophages were absent. However, isolates contained the same incomplete prophage (PHAGE_Escher_PA28 [Accession number: NC_041935]). The *bla*_CPHA3_ and *bla*_OXA-12_ genes were located on the chromosome, with >97% homology to *Aeromonas veronii* strain AVNIH1 (Accession no. CP047155.1) and *Aeromonas veronii* strain 17ISAe (Accession no. CP028133.1) (Table S3). The isolates contained 4–5 clustered, regularly interspaced, short palindromic repeat (CRISPR) arrays with no Cas element (Table 2). None of the strains harboured the restriction-modification (R-M) system.

### 3.4. Molecular Typing, Global Epidemiological and Phylogenomic Analysis

In silico determination of the sequence types (STs) of the isolates, using the *Aeromonas* MLST scheme resulted in an undescribed ST comprising new alleles for *gltA*_473, *groL*_445, *gyrB*_465, *metG*_470, *ppsA*_512 and *recA*_519. Allele sequences were submitted for curation and assigned to the new ST657 (Table 2). BURST (Based Upon Related Sequence Types) analysis identified the novel ST657 as a satellite clone (more distantly) with no single-, double nor triple-locus variants of global curated *A. veronii* STs.

**Figure 5:**
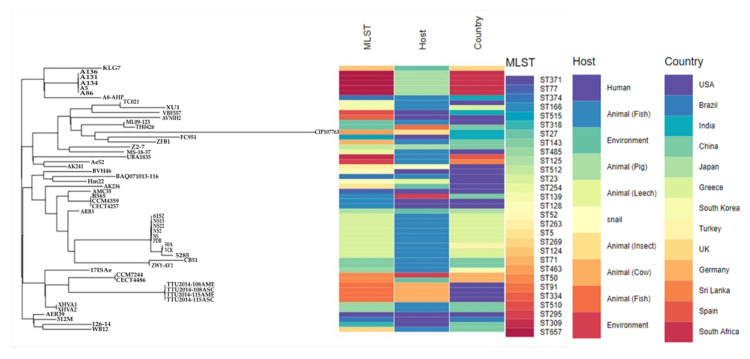
A global phylogenomic branch coupled with metadata of isolate (country; host (source) and WGS in silico sequence typing (STs)) showing the relationship between the study isolates (*n* = 5) and all deposited *A. veronii* strains (*n* = 49) from PATRIC database using Phandango. The colour codes differentiate the different countries, hosts, and sequence types.

The metadata analyses of the five isolates, together with the 49 global strains, showed the diversity of *A. veronii* sequence types (*n* = 29), country of origin (*n* = 13) and sources. The most prevalent sequence types were ST23 (*n* = 9), ST657 (*n* = 5, study isolates), ST91 (*n* = 4), ST166 (*n* = 3), ST485 (*n* = 3) and ST50 (*n* = 2) (Figure 5 and Table S4). The *A. veronii* isolates were from different sources, predominantly animal hosts (*n* = 38; mostly from fish sources [*n* = 25], pigs [*n* = 6] and cow [*n* = 4]), followed by humans (*n* = 10) and the environment (*n* = 6). The USA (*n* = 17), China (*n* = 10), Greece (*n* = 9), South Africa (*n* = 5) and India were the countries with the highest number of isolates deposited on the databases.

**Figure 6:**
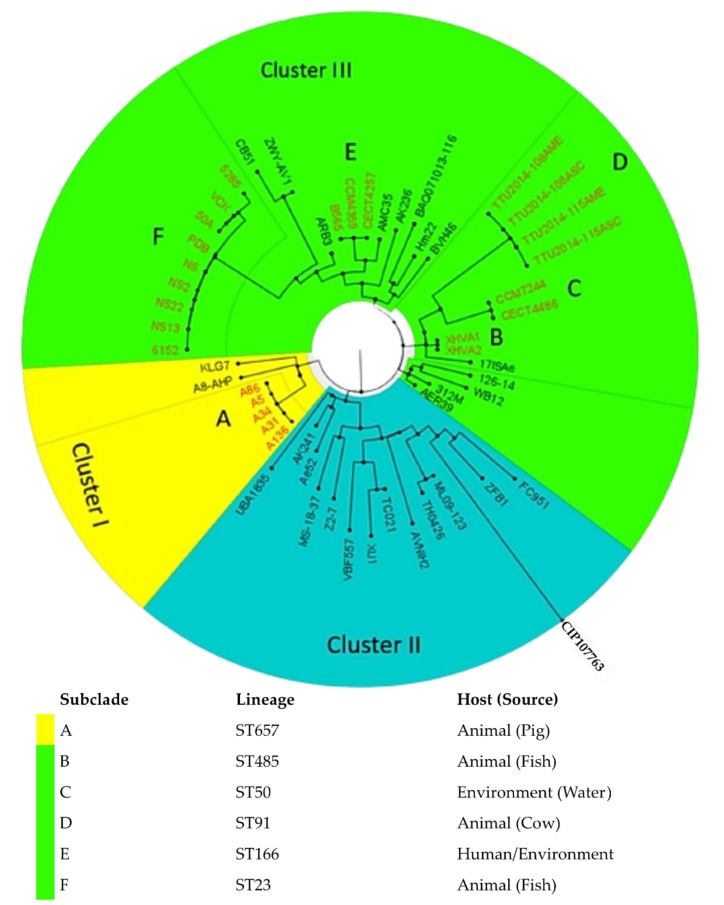
A circular cladogram of global *A. veronii* genomes depicting the association between isolates in three clusters (I, II and III). The KwaZulu-Natal (South Africa) isolates belonged to the smallest cluster I and were mainly related to international strains KLG7 (UK) and A8-AHP (India). Sub-clades (A-E) depicted a clustering of isolates, mainly according to sequence types/sources.

The phylogenomic analyses via the WGS SNP tree differentiated the global strains into three clusters (I, II and III) (Figure 5 and Figure 6). The study isolates were found in Cluster I with a 100% branch conservation and close lineage to two international stains, KLG7 (UK) and A8-AHP (India) from the environment and fish source, respectively. Cluster II contained strains (*n* = 14) which were mostly of animal origin (*n* = 11), except for three strains which were from humans (AVNIH2, VBF557 and FC951). Cluster III was the largest group and contained strains from diverse sources (humans, animals, and environment) (Figure 5 and Figure 6). Six highly conserved genetic subclades (A-E) were identified, which depicted a clustering of isolates mainly according to their sequence types (clonal lineages) and sources (Figure 6). Moreover, the results of the global phylogenetic tree demonstrated the complexity and diversity of *A. veronii* regarding geography, source, and clonality, with many unresolved clusters.

### 3.5. Pathogenic Potential and Putative Virulence Factors

**Figure 7:**
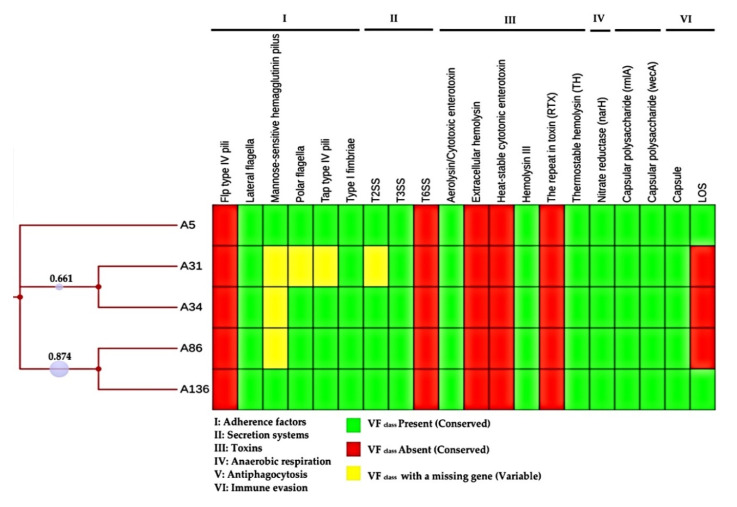
Heatmap generated with phylogenetic and distribution of virulence factors across the *A. veronii* isolates (*n* = 5). The colour coding depicts the presence (green), absence (red) and variability (yellow) of the virulence gene sets. The virulence factors are represented by Roman numerals I: adherence factors; II: secretion systems III toxins; IV; anaerobic respiration; V anti-phagocytosis and VI: immune evasion.

The mean pathogenicity score of 0.60 indicated the potential pathogenicity of *A. veronii* in humans and was found to match 30 pathogenic families. The whole virulome analysis predicted a total of 200 putative virulence-encoding genes belonging to six major virulence factor classes of *Aeromonas*, namely, adherence factor (lateral flagella, mannose-sensitive hemagglutinin pilus, polar flagella, tap type IV pili and type I fimbriae), secretion system (T2SS and T3SS), toxins (aerolysin/cytotoxic enterotoxin and hemolysin), anaerobic respiration (nitrate reductase), antiphagoctyosis (capsular polysaccharide) and immune evasion (capsule and LOS) with minor differences (Figure 7 and Table S5). A total of 195 conserved virulence factors were observed across the isolates.

## 4. Discussion

*Aeromonas* spp. are important human pathogens and colonizers and have also been increasingly isolated in animals (food-animals, wildlife, and companion animals), and the environment (soil, water, and air) globally. They, thus, have potential as One Health indicator bacteria for monitoring the spread of antibiotic-resistant bacteria between humans, animals and the environment [4,43,44,45,46,47]. However, information on this pathogen in food animals, using high-throughput technologies such as whole genome sequencing in Africa, is lacking. In this study, we describe for the first time in Africa, the comparative genomics of five *A. veronii* isolates recovered from pigs in South Africa. We also show the phylogenomic relationship between this novel strain and all globally deposited *A. veronii* genomes with complete metadata (country, sources and sequence types), as their incidence and geographic spread is vital to understand the evolution of this emerging pathogen which is on a global rise.

Analyses of the genomic data revealed a high degree of genomic synteny (>98%), suggesting a close association between the study isolates (Figure 1). All the isolates shared orthologous clusters (98.8%) with no unique gene cluster (Figure 2), indicating a relatively large set of core functions with low variable sections as well as a vast array of similar central networks (Figure 3) which are crucial for their survival in the microbial community [48]. The high degree of similarity between the isolates predicted by the different analyses also corroborated the novel clonal lineage (ST657), where the isolates possessed the same genetic make-up with low variation. For instance, mobilome analysis of the ST657 highlighted the lack of plasmids, integrons and intact prophages in this lineage. However, variability in chromosome-borne insertion sequence (IS) families was observed (Table 2 and Figure 4). More so, the isolates contained the same incomplete prophage (Escher_PA28) which did not harbor any resistance and virulence genes. A similar scattered IS pattern was previous reported in *A. veronii* by Tekedar et al. [20]. Further analyses of this clonal lineage (ST657) depicted a unique satellite-variant, implying that it was distantly related to global sequence types, hence does not have any ancestral linkage with the STs found in the *Aeromonas* MLST database.

Bacteria often accrue defence systems which offer protection against foreign DNA invasion and viral predation [49,50]. Regarding the CRISPR-Cas defence systems consisting of two main components, CRISPR array and associated genes (Cas), the *A*. *veronii* genomes encoded the CRISPR elements with no Cas system (Table 2). This shows that the genomes of the isolates contained short repeat clusters which have been implicated in bacterial adaptation strategies, ranging from immune evasion and tissue tropism to the modulation of environmental stress tolerance. This finding was similar to a study by Tekedar et al. [20], which confirmed the presence of CRISPR elements in all compared *A. veronii* isolates (*n* = 41), with only a few (*n* = 4) harbouring the Cas elements. This implies that the complete CRISPR-Cas systems are less prevalent in *A. veronii*, probably because of the lack of nucleotide biosynthesis capacity [51]. Moreover, the Cas systems were predominantly found in human isolates, suggesting the need for further studies to understand the CRISPR-Cas-mediated host interactions.

*A. veronii* has been reported in different food animals and products including pigs, chicken, cattle, sheep, buffalo and fish [10,46,52]. The consumption of undercooked/raw meat or meat products is an important route for human infection with *Aeromonas* spp. [4,10]. The carriage rate of carbapenem-resistant *A. veronii* isolates was 1.5%, which was comparable to the prevalence rate in food-producing animals in Europe (<1%) and in the lower range of the resistance reported in both Africa (2–26%) and Asia (1–15%) [53]. Although the overall prevalence of carbapenem-resistant *A. veronii* in food animals appears to be low, the transmission of these pathogens from food animals to their derived products could be a threat to consumers, supporting the transmission of resistant bacteria and their determinants between commensal and pathogenic microorganisms with unknown, but potentially severe, consequences for human health [53,54].

The resistance phenotypes corroborated the presence of bla_CPHA3_ and bla_OXA-12_ conferring resistance to imipenem and penicillin (ampicillin and amoxicillin) (Table 1 and Table 2). The Aeromonads, including *veronii* and *A. hydrophilia*, have been reported to harbour conserved resistance genes on their chromosome, conferring intrinsic resistance against these antibiotics [20,55,56]. More so, they often harbour genes that code for the production of β-lactamases such as class B metallo-β-lactamase, class C cephalosporinase, and class D penicillinases [3,5,41,42]. However, *Aeromonas* spp. are reported to be susceptible to monobactams, third-and fourth-generation cephalosporins, aminoglycosides, and fluoroquinolones, as found in this study [3].

The pathogenic potential (P_score_), with the probability ranging from 0 to 1, is used to predict the ability of bacteria to cause infection in humans [39,48]. This theoretical estimation of the pathogenic potential, using trained algorithms to differentiate between pathogenic or commensal strains, predicted a relatively higher average probability (P_score_ ≈ 0.60), suggesting that the clone (ST657) (Table 2) could be potentially pathogenic to humans. However, it originated from a non-human source, highlighting the role of *Aeromonas* spp. from food animal sources as potential human pathogens [4]. The global emergence of *Aeromonas* spp. in all One Health settings (human-animal-environment), makes it a potential One Health indicator organism.

Several insertion sequences were found in our isolates (Table 2 and Figure 4). Insertion elements are significant in the evolution of *Aeromonas* genome [57], contributing to its resistome and virulome through the incorporation of additional genes, genome reduction and rearrangement, gene decay and inactivation, and expansion of flanking regions [58,59]. The virulome analysis revealed the possession of a battery of determinants which play a significant role in their survival and pathogenesis, comparable to previously reported studies on *Aeromonas* spp. [5,20,55,60] and supporting the pathogenic potential of this pathogen. The ST657 clone contained an array of six putative virulence factor classes, which were mostly conserved across the isolates, suggesting that *A. veronii* relies heavily on these factors for host invasion, immune evasion, tissue damage, and competition in diverse ecological niches (Figure 7 and Table S5). Adherence factors were the most prevalent putative virulence determinants followed by secretion systems and toxins (Figure 7 and Table S5) in contrast to virulence factors possessed by *A. veronii* isolated from fish samples, where the secretion system and its components were found to be the most predominant [20]. This observation could imply that predominating virulence factors may be host-specific. Interestingly, differences in the virulome of the ST657 lineage were evident. Some of the isolates lacked specific genes within the sub-components of adherence factors, secretion systems and immune evasion virulence factors. For example, the T2SS sub-component of the secretion system, which is known for exporting hydrolytic enzymes and aids in the gut colonization [61,62], lacked the *exe*H gene in isolate A31. The expression of this putative virulome probably confers a competitive advantage, contributing to its success as a pathogen [48]. Moreover, the genomic detection of these virulence genes could aid in identifying targets for the development of novel vaccines for this emerging pathogen [63,64].

Global epidemiological comparison of deposited *A. veronii* genomes revealed the diverse nature of this pathogen regarding its host, clonal lineages, and geographical distribution (Figure 5 and Table S4). This diversity implies that the *A. veronii* can serve as good One Health indicator pathogen to understand and track the geographical spread of antibiotic resistance. Phylogenomic analysis depicted the clustering of group members from disparate geographies. Interestingly, the study isolates were closely related to strains from the UK and India, from different lineages (Figure 7), but not close enough to suggest import into South Africa from other countries. The small number of global deposited strains with insufficient metadata made it challenging to make much inference from the tree analysis on the transmission dynamics of this species, as there were many unresolved clusters. It is thus recommended that more studies be carried out in all the sectors (human, animal and environment) to harness the ability of genomics and bioinformatic analysis in making useful predictions about the dynamics of emerging pathogens in the One Health context.

## 5. Conclusions

The comparative genomics of *A. veronii* revealed the clonal dominance of the novel strain, ST657, isolated from South Africa. The genomic data presented lends useful insights into the pan-genome, resistome, defense system, virulome, pathogenic potential, clonal lineages, global dissemination, and phylogenetic relationship of this pathogen. To the best of our knowledge, this is the first comprehensive genomic analysis of *A. veronii* isolates in Africa and presents this species as a potential One Health indicator.

## Figures and Tables

**Table 1:** Susceptibility pattern of *A. veronii* isolates.

Antibiotics	Resistance Phenotypes ^1^				
	A5	A31	A34	A86	A136
Ampicillin (AMP)	R	R	R	R	R
Amoxicillin (AMX)	R	R	R	R	R
Amoxicillin-clavulanate (AMC)	I	S	S	S	I
Piperacillin-tazobactam (TZP)	S	S	S	S	R
Cefuroxime (CXM)	S	S	S	S	S
Cefotaxime (CTX)	S	S	S	S	S
Ceftriaxone (CRO)	S	S	S	S	S
Ceftazidime (CAZ)	S	S	S	S	S
Cefepime (FEP)	S	S	S	S	S
Cefoxitin (FOX)	S	S	S	S	S
Imipenem (IMI)	R	R	R	R	R
Meropenem (MER)	S	S	S	S	S

^1^ S = susceptible; I = intermediate; R = resistant.

**Table 2:** Summary of specimen source, sample type, and genotypic characteristics of the isolates.

Isolate	In Silico Typing	Defence Systems	Resistome	Mobilome ^2^	Pathogenicity ^3^
ID	MLST ^1^	CRISPR (Cas)		No. of Insertion Sequences	Score (Pathogenic Families)
A5	ST657	4 (0)	OXA-12, cphA3,	45	0.607 (30)
A31	ST657	5 (0)	OXA-12, cphA3,	68	0.607 (30)
A34	ST657	5 (0)	OXA-12, cphA3,	73	0.607 (30)
A86	ST657	4 (0)	OXA-12, cphA3,	43	0.603 (29)
A136	ST657	5 (0)	OXA-12, cphA3,	45	0.607 (30)

^1^ Isolates belonged to the same clone with sequence type (ST657). ^2^ None of the isolates possessed a restriction-modification (R-M) system, plasmids, integrons and intact prophage. The isolates contained the same incomplete prophage (PHAGE_Escher_PA28 [Accession number: NC_041935]. ^3^ Pathogenicity score predicted potential pathogenicity in humans.

## Supplementary Materials

The following are available online at https://www.mdpi.com/2076-2607/8/12/2008/s1, Table S1: Genomic features of the *A. veronii* isolates, Table S2: Distribution of selected functional categories in the *A. veronii* isolates, Table S3: Genetic environment of resistance genes found in the isolates, Table S4: Table showing the *A. veronii* isolates with full metadata (sequence type, host (source) and country of origin) retrieved from the PATRIC database, Table S5: Table of the virulence factor distribution in the 5 *A. veronii* genomes.
