# Supplementary material for: Comparative Pathogenomics of Aeromonas veronii from Pigs in South Africa: Dominance of the Novel ST657 Clone
Source: Microorganisms. 2020 Dec 16;8(12):2008. doi: 10.3390/microorganisms8122008 (PMC7765573; doi:10.3390/microorganisms8122008)
Supplement: Supplementary file 1 [file microorganisms-08-02008-s001.zip › microorganisms-1013302-sl/Supplementary data_Genes.docx]

**Table S1.** Genomic features of the *A. veronii* strains.

| **No.** | **Strain names** | **Size (Mb)** | **GC%** | **No. of contigs** | **N50(bp)** | **L50** | **No. of RNAs** | **Protein-coding sequences** | **Coverage%** | **Accession Number** |
| --- | --- | --- | --- | --- | --- | --- | --- | --- | --- | --- |
| 1 | A5 | 4.77 | 58.2 | 33 | 230041 | 7 | 92 | 4511 | 99 | VWTX00000000 |
| 2 | A31 | 4.64 | 58.5 | 84 | 114767 | 14 | 82 | 4380 | 102 | VWTW00000000 |
| 3 | A34 | 4.64 | 58.5 | 86 | 139859 | 13 | 86 | 4396 | 102 | VWTV00000000 |
| 4 | A86 | 4.64 | 58.5 | 42 | 206004 | 8 | 95 | 4359 | 102 | VWTT00000000 |
| 5 | A136 | 4.67 | 58.4 | 41 | 213730 | 7 | 94 | 4396 | 102 | VWTS00000000 |

**Table S2.** Distribution of selected functional categories in the *A. veronii* strains.

| **No.** | **Functional category of in silico predicted proteins** | 1. ***veronii* strains** | | | | |
| --- | --- | --- | --- | --- | --- | --- |
|  |  | **A5** | **A31** | **A34** | **A86** | **A136** |
| 1 | Cofactors, Vitamins, Prosthetic Groups, Pigments | 167 | 167 | 168 | 167 | 167 |
| 2 | Cell Wall and Capsule | 28 | 28 | 28 | 28 | 28 |
| 3 | Potassium Metabolism | 9 | 9 | 9 | 9 | 9 |
| 4 | Miscellaneous | 9 | 9 | 9 | 9 | 9 |
| 5 | Membrane Transport | 137 | 137 | 136 | 136 | 136 |
| 6 | Iron acquisition and metabolism | 11 | 11 | 11 | 11 | 11 |
| 7 | RNA Metabolism | 51 | 51 | 51 | 51 | 51 |
| 8 | Nucleosides and Nucleotides | 97 | 97 | 98 | 97 | 98 |
| 9 | Protein Metabolism | 220 | 219 | 222 | 224 | 227 |
| 10 | Cell Division and Cell Cycle | 6 | 6 | 6 | 6 | 6 |
| 11 | Regulation and Cell signalling | 34 | 34 | 34 | 34 | 34 |
| 12 | Secondary Metabolism | 4 | 4 | 4 | 4 | 4 |
| 13 | DNA Metabolism | 102 | 98 | 99 | 98 | 98 |
| 14 | Fatty Acids, Lipids and Isoprenoids | 59 | 59 | 60 | 59 | 59 |
| 15 | Nitrogen Metabolism | 39 | 39 | 40 | 39 | 39 |
| 16 | Dominance and Sporulation | 3 | 3 | 3 | 3 | 3 |
| 17 | Respiration | 108 | 108 | 110 | 108 | 108 |
| 18 | Stress Response | 70 | 71 | 70 | 70 | 71 |
| 19 | Metabolism of Aromatic Compounds | 10 | 10 | 10 | 10 | 10 |
| 20 | Amino Acids and Derivatives | 353 | 353 | 353 | 353 | 353 |
| 21 | Sulfur Metabolism | 7 | 7 | 7 | 7 | 7 |
| 22 | Phosphorus Metabolism | 35 | 35 | 35 | 35 | 35 |
| 23 | Carbohydrate Metabolism | 276 | 277 | 276 | 276 | 276 |
| 24 | Virulence, Disease and Defense | 40 | 46 | 45 | 44 | 45 |
| 25 | Motility and chemotaxis | 122 | 122 | 122 | 122 | 122 |

**Table S3.** Genetic environment of resistance genes found in the isolates.

| **Strain (MLST)** | **Contig** | **Resistance gene/s**  **(position on contig)** | **Mobile genetic elements** | **Plasmid/Chromosomal sequence with the closet nucleotide homology**  **(accession number)** |
| --- | --- | --- | --- | --- |
| A5 (ST657) | 14 | bla_CPHA3_ (12605..13369) | None | *Aeromonas veronii* strain AVNIH1 chromosome (CP047155.1) |
|  | 17 | bla_OXA-12_ (11009..11781) | None | *Aeromonas veronii* strain 17ISAe chromosome (CP028133.1) |
| A31 (ST657) | 9 | bla_CPHA3_ (12605..13369) | None | *Aeromonas veronii* strain AVNIH1 chromosome (CP047155.1) |
|  | 29 | bla_OXA-12_ (11009..11781) | None | *Aeromonas veronii* strain 17ISAe chromosome (CP028133.1)) |
| A34 (ST657) | 9 | bla_CPHA3_ (12605..13369) | None | *Aeromonas veronii* strain AVNIH1 chromosome (CP047155.1) |
|  | 21 | bla_OXA-12_ (11009..11781) | None | *Aeromonas veronii* strain 17ISAe chromosome (CP028133.1) |
| A86 (ST657) | 5 | bla_CPHA3_ (274887..275651) | None | *Aeromonas veronii* strain AVNIH1 chromosome (CP047155.1) |
|  | 18 | bla_OXA-12_ (11009..11781) | None | *Aeromonas veronii* strain 17ISAe chromosome (CP028133.1) |
| A136 (ST657) | 15 | bla_CPHA3_ (12605..13369) | None | *Aeromonas veronii* strain AVNIH1 chromosome (CP047155.1) |
|  | 16 | bla_OXA-12_ (11009..11781) | None | *Aeromonas veronii* strain 17ISAe chromosome (CP028133.1) |

**Table S4.** Table showing the *A. veronii* isolates with full metadata (sequence type, host (source) and country of origin) retrieved from the PATRIC database.

| **Strain** | **Country** | **Host** | **MLST** |
| --- | --- | --- | --- |
| AER39 | USA | Human | ST371 |
| AMC35 | USA | Human | ST77 |
| 312M | Brazil | Human | ST374 |
| CECT4257 | USA | Human | ST166 |
| CCM4359 | USA | Human | ST166 |
| FC951 | India | Human | ST515 |
| 126-14 | China | Human | ST318 |
| ML09-123 | USA | Animal (Fish) | ST27 |
| ZWY-AV1 | China | Animal (Fish) | ST143 |
| XHVA1 | China | Animal (Fish) | ST485 |
| ARB3 | Japan | Environment (Pond) | ST125 |
| Z2-7 | China | Animal (Pig) | ST512 |
| XHVA2 | China | Animal (Fish) | ST485 |
| VCK | Greece | Animal (Fish) | ST23 |
| PDB | Greece | Animal (Fish) | ST23 |
| 5285 | Greece | Animal (Fish) | ST23 |
| NS | Greece | Animal (Fish) | ST23 |
| 17ISAe | South Korea | Animal (Fish) | ST485 |
| MS-18-37 | USA | Animal (Fish) | ST254 |
| NS2 | Greece | Animal (Fish) | ST23 |
| 50A | Turkey | Animal (Fish) | ST23 |
| 6152 | Greece | Animal (Fish) | ST23 |
| XU1 | Greece | Animal (Fish) | ST139 |
| TC021 | USA | Animal (Fish) | ST139 |
| Hm22 | USA | Animal (Leech) | ST128 |
| BVH46 | USA | Human | ST52 |
| AK241 | USA | Animal (snail) | ST263 |
| BAQ071013-116 | USA | Animal (Fish) | ST374 |
| AK236 | USA | Environment (Lake) | ST5 |
| WB12 | China | Animal (Fish) | ST269 |
| NS22 | Greece | Animal (Fish) | ST23 |
| NS13 | Greece | Animal (Fish) | ST23 |
| KLG7 | UK | Environment (River) | ST124 |
| A8-AHP | India | Animal (Fish) | ST374 |
| ZFB1 | China | Animal (Fish) | ST71 |
| CIP107763 | India | Animal (Insect) | ST463 |
| CECT4486 | Germany | Environment | ST50 |
| TTU2014-108AME | USA | Animal (Cow) | ST91 |
| TTU2014-108ASC | USA | Animal (Cow) | ST91 |
| TTU2014-115AME | USA | Animal (Cow) | ST91 |
| TTU2014-115ASC | USA | Animal (Cow) | ST91 |
| TH0426 | China | Animal (Fish) | ST27 |
| CB51 | China | Animal (Fish) | ST143 |
| AVNIH2 | USA | Human | ST334 |
| VBF557 | India | Human | ST510 |
| Ae52 | Sri Lanka | Animal (Fish) | ST295 |
| CCM7244 | Germany | Environment (Water) | ST50 |
| UBA1835 | Spain | Animal (Fish) | ST309 |
| B565 | China | Environment (Pond) | ST166 |
| A5 * | South Africa | Animal (Pig) | ST657 |
| A31 * | South Africa | Animal (Pig) | ST657 |
| A34 * | South Africa | Animal (Pig) | ST657 |
| A86 * | South Africa | Animal (Pig) | ST657 |
| A136 * | South Africa | Animal (Pig) | ST657 |

NB- *-🡪 Study isolates.
